# A Novel BBS9 Mutation Identified via Whole-Exome Sequencing in a Chinese Family with Bardet-Biedl Syndrome

**DOI:** 10.1155/2021/4514967

**Published:** 2021-10-15

**Authors:** Yue Zhang, Manhong Xu, Minglian Zhang, Guoxing Yang, Xiaorong Li

**Affiliations:** ^1^Tianjin Key Laboratory of Retinal Functions and Diseases, Tianjin Branch of National Clinical Research Center for Ocular Disease, Eye Institute and School of Optometry, Tianjin Medical University Eye Hospital, Tianjin 300384, China; ^2^Hebei Provincial Key Laboratory of Ophthalmology, Hebei Provincial Eye Institute, Hebei Provincial Eye Hospital, Xingtai, Hebei 054001, China

## Abstract

Bardet-Biedl syndrome (BBS) is a genetically heterogeneous disorder characterized by polydactyly, obesity, rod-cone dystrophy, and mental retardation. Twenty-one genes have been identified as causing BBS. This study collected a BBS pedigree from two patients and performed whole-exome sequencing on one patient. We identified a novel homozygous variant c.1114C>T (p.Q372X) in the BBS9 of the two siblings. This variant was confirmed and completely cosegregated with the disease of this family by Sanger sequencing. We report a novel homozygous variant c.1114C>T in the BBS9 gene in a Chinese family.

## 1. Introduction

Bardet-Biedl syndrome (BBS) is a rare multisystem disorder exhibiting clinical and genetic heterogeneity. It is characterized by childhood-onset rod-cone dystrophy, postaxial polydactyly, truncal obesity, intellectual disability, male hypogonadotropic hypogonadism, complex female genitourinary malformations, and renal dysfunction. The frequency of BBS is 1 : 160000 in the northern European population, and it is more common in the Middle East. The incidence of BBS in the Chinese population has not yet been reported. BBS is inherited in an autosomal recessive manner. Currently, twenty-one genes have been reported to cause BBS. Most BBS-related genes are involved in ciliary function. The proteins encoded by the BBS gene family members are structurally diverse, and the similar phenotypes exhibited by mutations in BBS gene family members are likely due to their shared roles in cilia formation and function. BBS proteins localize to the basal bodies, ciliary axonemes, and pericentriolar regions of cells. BBS proteins are also involved in intracellular trafficking via microtubule-related transport [1–9].

In this study, we described the clinical features and molecular genetic results of a Chinese family with BBS.

## 2. Materials and Methods

### 2.1. Patients and Clinical Data

The patients in this study were from Hebei Province, China. Clinical examination and peripheral blood collection were done at Hebei Provincial Eye Hospital. This study followed the tenets of the Declaration of Helsinki, and it was approved by the Ethics Committee of Hebei Provincial Eye Hospital. The methods were carried out in accordance with the approved guidelines. All participants gave their written informed consent. The proband and his family members received ocular examinations, including best-corrected visual acuity (BCVA), indirect ophthalmoscopy, fundus photographs, and spectrum-domain optical coherence tomography (OCT; Heidelberg Engineering, Heidelberg, Germany). The proband also received a general physical examination.

### 2.2. Genomic DNA Extraction and Exome Sequencing

Genomic DNA, which was extracted from the proband, was purified from peripheral blood leukocytes using the QIAamp blood kit, according to the manufacturer's protocols (Qiagen, Hilden, Germany). WES was performed by ruibiotech, China. Exons of DNA samples were captured using the in-solution SureSelect Target Enrichment System (Agilent, Human All Exon Kits v2) (Agilent Technologies, Inc., Santa Clara, CA, USA), followed by a paired-end high-throughput sequencing on reads of 75 bp using Illumina HiSeq2000 (Illumina Inc., San Diego, CA, USA). Image analysis was performed with default parameters of Illumina RTA v1.12.4 pipeline. Base calling was performed with CASAVA 1.8.2 (Illumina).

### 2.3. Alignment, Variant Calling, and Mutation Detection

The sequence reads were aligned with the human genome reference sequence (University of California Santa Cruz, human genome assembly 19 (UCSC hg19)) using the Burrows-Wheeler transform algorithm. Variants (single-nucleotide variants (SNVs) and short insertion-deletion variants (Indels)) were called using SAMTools software (http://samtools.sourceforge.net/) with reference to public databases, dbSNP and 1000 Genomes. SNVs were filtered for a minimum Phred quality score of 30, allowing 99.9% base call accuracy. The analysis was performed with preference given to variants located in the 21 genes causing BBS. We first removed nonexonic and synonymous variants. This was followed by removing common variants (i.e., minor allele frequency > 0.01) reported in public databases. SIFT, PolyPhen-2, and MutationTaster were used to predict the functional effects of the selected variants. All missense variants predicted to be benign were removed from the list of variants. Among the prioritized variants, those harboring truncating mutations, or mutations predicted to be damaging, were considered the most promising candidates. PubMed and OMIM were reviewed for previous publications regarding candidate genes and functional and expression data.

### 2.4. Sanger Sequencing Validation and Segregation Analysis

Potential pathogenic variants detected with WES were validated using Sanger sequencing. DNA from family members was examined to investigate the cosegregation of potentially pathogenic variants. Sanger sequencing was performed using standard polymerase chain reaction (PCR) amplification procedures. The following primers of exon 10 for the BBS9 gene were used: forward primer 5′-tggtgtgttgatgctgaattga-3′ and reverse primer 5′-agacataaagaccttgtgtgct-3′.

## 3. Results

### 3.1. Clinical Findings

**Figure 1:**
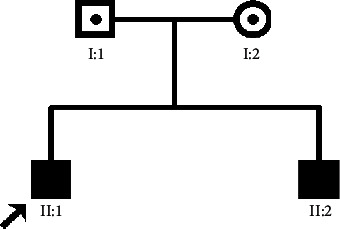
Pedigree of the Bardet-Biedl syndrome family.

**Figure 2:**
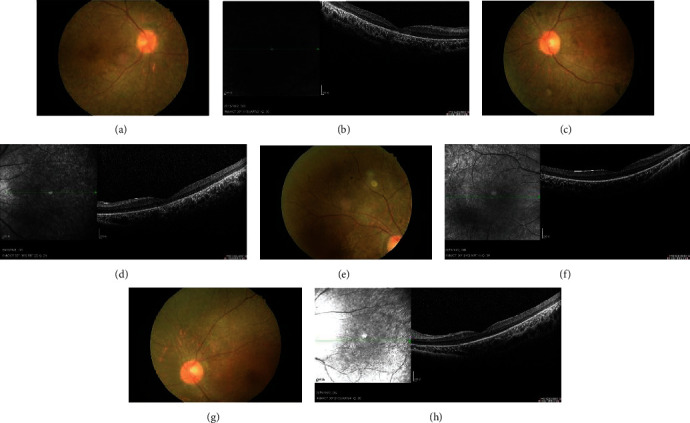
Fundus imaging of the Bardet-Biedl syndrome family. Fundus photograph and optical coherence tomography. Fundus photograph showed vascular attenuation, diffuse RPE atrophic changes, and retinal pigmentation in patient I (a, c) and patient II-1 (e, g). OCT showed atrophic changes in the outer layer of the retina in patient I (b, d) and patient II-2 (f, h).

**Figure 3:**
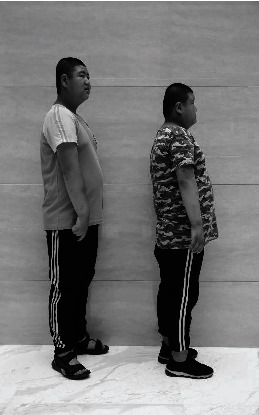
Photography of the patients. The patients showed obesity compared with people of the same age.

**Figure 4:**
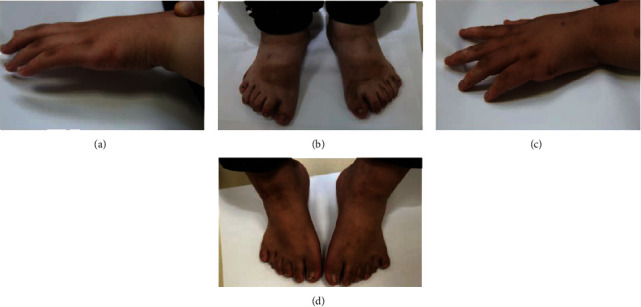
The hands and toes of the affected participants from the Bardet-Biedl syndrome family. Physical examination, six toes, and surgical scars were observed in patient II-1 (a, b) and patient II-2 (c, d).

The patients II-1 and II-2 in the family showed typical BBS on examination (Figure 1). Two patients showed polydactyly, obesity, rod-cone dystrophy, and mental retardation. The patients experienced visual problems throughout the entire day since early childhood. Ocular examinations showed that the BCVA of patient II-1 was 0.1/0.1, with refractions of +3.0 D for both eyes. Meanwhile, patient II-2 had a BCVA of 0.25/0.25, with refractions of +1.0 D for both eyes. Ophthalmoscopic examination displayed some evidence of rod-cone dystrophy (Figure 2), including attenuation of the retinal vessels and waxy atrophy of the optic disc. Her right eye showed more severe signs. However, few pigmentary deposits were noted in the peripheral retina of both eyes. Instead, retinal pigment epithelium (RPE) mottling and choroidal sclerosis-type changes were seen. SD-OCT revealed significant bilateral structural damage in the macula (Figure 2), including extensive thinning of the neuroretina, almost complete absence of the inner/outer segment (IS/OS) junctions which were partially preserved in the fovea, and dystrophy of the RPE. Patient II-1 had a bodyweight of 90.5 kg, height of 169 cm, and body mass index (BMI) of 31.69 kg/m^2^. Patient II-2 had a bodyweight of 76 kg, height of 155 cm, and BMI of 31.63 kg/m^2^ (Figure 3). On physical examination, the two patients had six toes on both feet. Patient II-1 had six fingers on both hands since birth. An extra finger was removed at childhood, and surgical scars were observed on both hands. Patient II-2 had six fingers on the left hand since birth. An extra finger was removed at childhood, and a surgical scar was observed on his left hand (Figure 4). The patients' intelligence was estimated based on their social performance. The patients showed poor understanding and language communication difficulties, which were also reported by their parents. Therefore, the patients were considered mentally retarded.

The patients' parents had no physical anomalies, and their ocular examination results were normal. Other members of both sides of the family did not have similar diseases or symptoms.

### 3.2. Mutation Analysis

**Figure 5:**
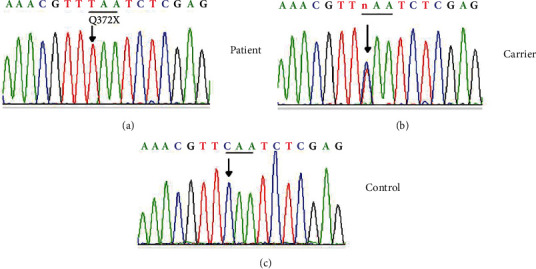
DNA sequences of *BBS9* in the affected individual, carrier, and control. A homozygous c.1114C>T (p.Q372X) mutation in the BBS9 was identified in the patient.

WES was performed in patient II-1 of the family. Bioinformatics analysis revealed a novel homozygous variant c.1114C>T (p.Q372X) in the BBS9 of the two siblings(Figure 5). This variant was confirmed and completely cosegregated with the disease of the family by Sanger sequencing. The c.1114C>T mutation was predicted to cause a loss of function.

## 4. Discussion

BBS is a kind of ciliopathies which caused defects in the structure and/or function of cilia. BBS had some overlapping phenotypes and genotypes with other ciliopathies, such as Alström syndrome, McKusick-Kaufman syndrome, Joubert syndrome, Meckel-Gruber syndrome, or polycystic kidney disease. BBS is clinically and genetically heterogeneous, with various primary and secondary clinical manifestations. There is no clear genotype-phenotype correlation [10].

The Bardet-Biedl syndrome protein complex (BBSome) is an octameric complex that transports membrane proteins into the primary cilium signaling organelle in eukaryotes and is implicated in human disease. The loss of any protein from the BBSome complex makes it lose its functionality of the cilium. The BBS9 protein is one of the eight BBSome components. The protein is composed of four structured domains, including an N-terminal *β*-propeller domain, *γ*-adaptin ear (GAE) *β*-sandwich domain, mixed *α*/*β* platform domain, and C-terminal *α*-helical domain. The p.Q372X was predicted to stimulate a premature stop codon and lead to the deletion of three domains at the C-terminal of BBS9 protein [11].

Fifty mutations in BBS9 have previously been reported (one in our study). The mutations included twenty-four missense/nonsense, eight splicing, sixteen deletion/insertion, and one complex rearrangement [12–14].

The patients' parents are nonconsanguineous, but the mutation analysis indicated that this family might be inbred. In previous reports, the founder effect of mutation c.299delC (p.Ser100Leufs∗24) in the BBS9 gene was observed in consanguineous Pakistani families [15, 16].

## 5. Conclusions

In conclusion, we found a novel homozygous variant c.1114C>T in BBS9 of an autosomal recessive Bardet-Biedl syndrome family. These findings expanded the mutation spectrum of BBS9.

## Data Availability

Data are available on request.
